# Synthetic CT Generation Based on T2 Weighted MRI of Nasopharyngeal Carcinoma (NPC) Using a Deep Convolutional Neural Network (DCNN)

**DOI:** 10.3389/fonc.2019.01333

**Published:** 2019-11-29

**Authors:** Yuenan Wang, Chenbin Liu, Xiao Zhang, Weiwei Deng

**Affiliations:** ^1^Department of Radiation Oncology, National Cancer Center/National Clinical Research Center for Cancer/Cancer Hospital & Shenzhen Hospital, Chinese Academy of Medical Sciences and Peking Union Medical College, Shenzhen, China; ^2^Department of Mechanics and Aerospace Engineering, Southern University of Science and Technology, Shenzhen, China

**Keywords:** synthetic CT (sCT), magnetic resonance imaging (MRI), deep learning, convolutional neural network (CNN), nasopharyngeal carcinoma (NPC), U-net

## Abstract

**Purpose:** There is an emerging interest of applying magnetic resonance imaging (MRI) to radiotherapy (RT) due to its superior soft tissue contrast for accurate target delineation as well as functional information for evaluating treatment response. MRI-based RT planning has great potential to enable dose escalation to tumors while reducing toxicities to surrounding normal tissues in RT treatments of nasopharyngeal carcinoma (NPC). Our study aims to generate synthetic CT from T2-weighted MRI using a deep learning algorithm.

**Methods:** Thirty-three NPC patients were retrospectively selected for this study with local IRB's approval. All patients underwent clinical CT simulation and 1.5T MRI within the same week in our hospital. Prior to CT/MRI image registration, we had to normalize two different modalities to a similar intensity scale using the histogram matching method. Then CT and T2 weighted MRI were rigidly and deformably registered using intensity-based registration toolbox *elastix* (version 4.9). A U-net deep learning algorithm with 23 convolutional layers was developed to generate synthetic CT (sCT) using 23 NPC patients' images as the training set. The rest 10 NPC patients were used as the test set (~1/3 of all datasets). Mean absolute error (MAE) and mean error (ME) were calculated to evaluate HU differences between true CT and sCT in bone, soft tissue and overall region.

**Results:** The proposed U-net algorithm was able to create sCT based on T2-weighted MRI in NPC patients, which took 7 s per patient on average. Compared to true CT, MAE of sCT in all tested patients was 97 ± 13 Hounsfield Unit (HU) in soft tissue, 131 ± 24 HU in overall region, and 357 ± 44 HU in bone, respectively. ME was −48 ± 10 HU in soft tissue, −6 ± 13 HU in overall region, and 247 ± 44 HU in bone, respectively. The majority soft tissue and bone region was reconstructed accurately except the interface between soft tissue and bone and some delicate structures in nasal cavity, where the inaccuracy was induced by imperfect deformable registration. One patient example was shown with almost no difference in dose distribution using true CT vs. sCT in the PTV regions in the sinus area with fine bone structures.

**Conclusion:** Our study indicates that it is feasible to generate high quality sCT images based on T2-weighted MRI using the deep learning algorithm in patients with nasopharyngeal carcinoma, which may have great clinical potential for MRI-only treatment planning in the future.

## Introduction

There is an emerging interest in applying magnetic resonance imaging (MRI) during radiation treatment (RT) (1, 2). This is mainly because MRI can provide superior soft tissue contrast without ionizing radiation. MRI offers more consistent and accurate target delineation in head and neck cancers, brain tumors, sarcomas, and tumor sites in the abdomen and pelvis (3–6). It has been reported that applying MRI to RT has great benefits to improve radiation dosimetry and to increase therapeutic ratio, such as reducing toxicity to critical organs and enabling dose escalation to tumor sites to achieve survival gains (4, 7). In addition, not only anatomical but also functional information can be obtained non-invasively using MRI, which makes MRI suitable for quantitative and longitudinal evaluation of treatment response (8–10). Therefore, MRI integrated with the conventional CT-sim in RT planning has become an essential step in modern RT process (1–3).

As we know, nasopharyngeal carcinoma (NPC) is a common malignancy in Southeast Asia. Integrating MRI to RT in patients with NPC can be especially helpful due to its relatively complicated target structures and surrounding critical normal tissues. Accurate delineation of critical structures and tumors in NPC may not only help patients gain survival but also improve life quality. However, there are multiple challenges in integrating MRI to clinical RT. The acquisition time of MRI pulse sequences is typically much longer than CT, since the MRI scanning protocol generally includes not only localizer, T1 weighted, T2 weighted, diffusion weighted imaging (DWI) but also dynamic contrast enhanced (DCE) sequences. Also, parameters of MRI pulse sequences such as bandwidth, TR, TE and the receiver coils need to be manipulated based on patients' anatomical sites or pathological examinations. MRI in general is more technically challenging to radiation physicists and physicians compared to CT. Hence, MRI technologists may need more time to adjust complex parameters or to optimize coils during an MRI scan (11). Secondly, MRI is inherently susceptible to motion artifact and geometric distortion (1, 2, 11, 12). For example, the geometrical uncertainty of ~2 and 2–3 mm was observed for the brain and pelvic sites, respectively (13, 14). Such systematic errors can lead to RT target miss and compromise local control.

Another well-known challenge lies in the conversion of electron density or HU values in synthetic CT based on MR images. CT images can be used for radiation treatment planning is because they can be directly scaled to photon attenuation map. However, MRI does not provide such information (11, 12). Currently there are three methods of mapping HU based on the intensity of MR images (15, 16): atlas-based (17), voxel-based (18), and hybrid type (19). The atlas-based method of producing synthetic CT images may require CT to MRI registration where CT and MRI atlas scan pair can correspond anatomically (17). In contrast, voxel-based method is focused on using voxel by voxel mapping based on intensity or spatial location of the MRI images acquired from different MRI pulse sequences (18). The hybrid method combines atlas- and voxel-based methods, where deformable registration from the atlas-based method and local pattern recognition from the voxel-based method are applied to obtain attenuation information in the MR images. From this point of view, our proposed deep learning method where both registration and voxel-by-voxel patterns are learned through U-net, can be considered as the hybrid method.

In fact, machine learning and deep learning have been applied to many medical fields including radiation oncology (20), which has main components of data, model, cost or loss of the model, and model optimizer. Topics of how to apply and what are the challenges of machine learning, neural networks, and artificial intelligence (AI) to the clinical RT process have been discussed previously on the red journal (21). Here we aim to apply deep learning algorithms such as the U-net convolution neural network (CNN) approach to convert T2-weighted MRI to synthetic CT.

## Materials and Methods

**Figure 1:**
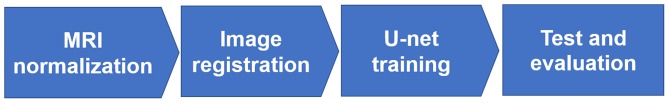
The workflow diagram of generating synthetic CT from T2-weighted MRI using U-net.

To convert the T2-weighted MRI to synthetic CT images, there were four major steps in our method illustrated in Figure 1: (1) MR image normalization into the similar intensity scale; (2) voxel-based rigid and deformable registration for CT and MRI; (3) U-net model training with 2/3 datasets; (4) U-net model testing with the rest 1/3 datasets and evaluation of the synthetic CT images.

### Data Acquisition

Thirty-three nasopharyngeal carcinoma (NPC) patients were retrospectively selected for this study with the approval of our hospital's internal review board (IRB). All patients underwent CT simulation in the head-first supine position with the Civco 5-point head, neck and shoulder mask on a GE Discovery CT scanner (Milwaukee, WI, USA) prior to RT planning with resolution of 512 × 512, slice thickness of 2.5 mm, 120 kVp and 300 mAs. Within the same week of CT acquisitions, diagnostic MRI was obtained using 1.5 T Siemens Avanto MRI scanner (Erlangen, Germany) in our hospital, where T2 weighted MRI was acquired using fat-saturated (FS) turbo spin echo (TSE) with resolution of 256 × 256 and slice thickness of 5 mm.

### Image Preprocessing

Prior to the rigid and deformable registration between T2 weighted MRI and CT images, we had to normalize the two imaging sets of different modalities to a similar intensity scale using the histogram matching method (Figure 1's first step: MRI normalization). Although lacking of a normalized intensity scale of MRI had no impact on clinical diagnosis provided by radiologists, it would influence the quality of image registration and deep learning, which highly depended on the similarity of image intensity between MRI and CT to achieve high-quality results. We used histogram matching method, which was independent of patients' image sets and specific brands of the MRI scanner used (22). In our study, the normalization process took account of all the NPC patients' samples by identifying 10 decile landmarks in the histogram of each MR image and calculated the mean values of each landmark as the standard scale. It was used to transform the MR images of the same protocol and body region to the standard scale (23).

To conduct rigid and deformable registration of the MRI and CT imaging modalities, we used an open source image registration package called *elastix* (version 4.9) (24, 25), where the traditional iterative intensity-based image registration method was applied. For all NPC patients, the rigid image registration was performed followed by deformable registration. In the rigid registration, multi-resolution registration method was used, and the optimizer was adaptive stochastic gradient descent. In the deformable registration, multi-metric and multi-resolution registration method was used with advanced Mattes mutual information as the similarity metrics and transform bending energy penalty for smooth displacement (26) (Figure 1's second step: Image registration).

**Figure 2:**
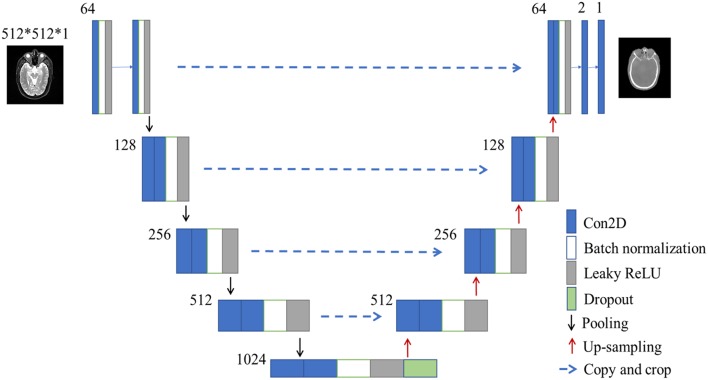
The architecture of U-net used in this study. The size of input images is 512^*^512^*^1 pixels. Blue box is multi-channel feature map, and the number on the top-left of the box is channel number. White box is batch normalization. Gray box is leaky ReLU. Green box is dropout module.

After image normalization and image registration steps described as the above, a U-net deep learning method was developed to generate synthetic CT from T2-weighted MRI using 23 convolutional layers of CNN, shown in Figure 2. To train and evaluate the U-net model, the 33 patients' dataset were randomly divided into two groups: 23 were used as the training set (~2/3 of the total datasets) and the rest 10 were used as the test set (~1/3 of the total datasets) (Figure 1's third and fourth steps).

### U-Net as a Deep Learning Algorithm

The U-net CNN structure consists of a contracting path and an expansive path (27), shown in Figure 2. The contracting path follows the typical architecture of a convolutional network. It consists of the repeated application of two 3 × 3 convolutions (unpadded convolutions), each followed by a rectified linear unit (ReLU) and a 2 × 2 max pooling operation with a stride of 2 for down-sampling. At each down-sampling step, we doubled the number of feature channels.

In contrast to the contracting path, the expansive path is composed of an up-sampling of the feature map followed by a 2 × 2 convolution (i.e., “up-convolution”) that halved the number of feature channels, a concatenation with the correspondingly cropped feature map from the contracting path, and two 3 × 3 convolutions, each followed by a ReLU. The cropping is necessary due to the loss of border pixels in every convolution. At the final layer, a 1 × 1 convolution was used to map each 64-component feature vector to the desired number of classes. In the final layer, a convolution was used to map the feature to the desired value, which was the intensity of the synthetic CT. Therefore, in the expansive path, a large amount of image features was used to reconstruct a new image of the same size as the input one. The implementation of our U-net was shown in Figure 2.

Here we used batch normalization and leaky ReLU in our network, which was different from the classical U-net (27). Our U-net was developed in Keras framework which was a high-level neural network API with Tensorflow as the backend. In total, the U-net network in our study had 23 convolutional layers. To allow a seamless tiling of the output segmentation map, we also selected the input tile size such that all 2 × 2 max-pooling operations were applied to a layer with an even x- and y-size.

### Evaluation

The 33 NPC patients were randomly divided into two groups: 23 as the training set and 10 as the test set. The U-net model described in the previous section was trained through feeding MRI and CT images from the training set into the neural network. The synthetic CTs were generated using the trained model for the test set.

To visually inspect the difference between true CT and synthetic CT, difference maps were generated. The pixel intensity of the difference map was the absolute difference between real CT and synthetic CT. Darker region in the difference map indicated smaller errors of CT values or HU number in the region of synthetic CT, and vice versa.

The mean absolute error (MAE) and mean error (ME) were used to quantify the absolute difference and mean difference within the body, respectively. The body masks were generated using OTSU's thresholding method and morphological operations (28, 29).

(1) $$\begin{array}{l} MAE=\frac{1}{n}\overset{n}{\underset{i=1}{\sum }}|CT\left(i\right)-sCT\left(i\right)| \end{array}$$

(2) $$\begin{array}{l} ME=\frac{1}{n}\overset{n}{\underset{i=1}{\sum }}\left(CT\left(i\right)-sCT\left(i\right)\right) \end{array}$$

where *n* is the total number of pixels within the body outline. *CT(i)* is the *i*th pixel in real CT image, and *sCT(i)* is the *i*th pixel in the synthetic sCT.

To further evaluate the accuracy of synthetic CTs in different tissues, the threshold of 300 HU was used on the true CT images to separate the bone and soft tissues. The MAEs and MEs in bone and soft tissues were calculated, respectively.

## Results

### Comparison of True CT and Synthetic CT Images

**Figure 3:**
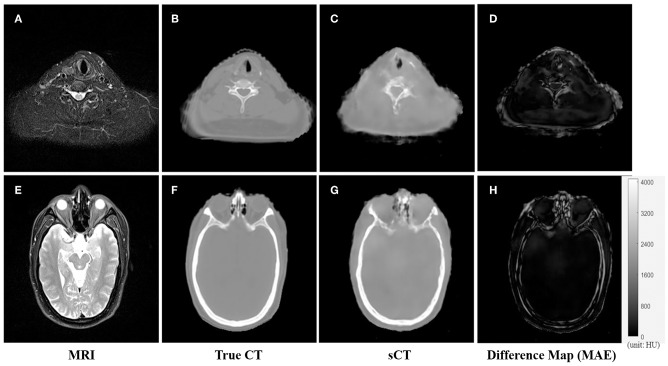
The comparison of CT and synthetic CT for two representative MR images. **(A)** MR image in the neck region; **(B)** real CT image aligned with **(A)**; **(C)** synthetic sCT converted from MR image **(A)**; **(D)** difference map between **(B,C)**; **(E)** MR image in the head region; **(F)** real CT image; **(G)** synthetic sCT image; **(H)** difference map between **(F,G)**. Gray bar in **(H)** indicated the mapping from CT number to gray scale in the difference maps.

An example of the T2-weighted MRI, true CT-sim, synthetic CT, and MAE differences in the axial view of two representative slices was shown in the first to fourth column in Figure 3. Soft tissues in the synthetic CT (Figures 3C,G) had similar intensities as the true CT (Figures 3B,F). The major difference between true CTs and synthetic CTs was in the air-bone and bone-soft tissue interface (Figures 3D,H: the MAE map).

**Figure 4:**
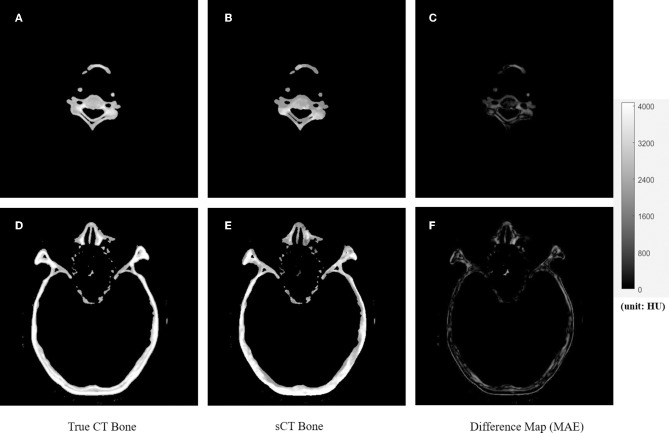
The comparison of true CT and synthetic CT for bone. The first column is true CT images. The second column is the synthetic CT images. The third column is the difference maps. **(A–C)** Showed bone in the neck region. **(D–F)** Showed bone in the head and nasal region. Gray bar indicated the mapping from CT number to gray scale in the difference maps.

**Figure 5:**
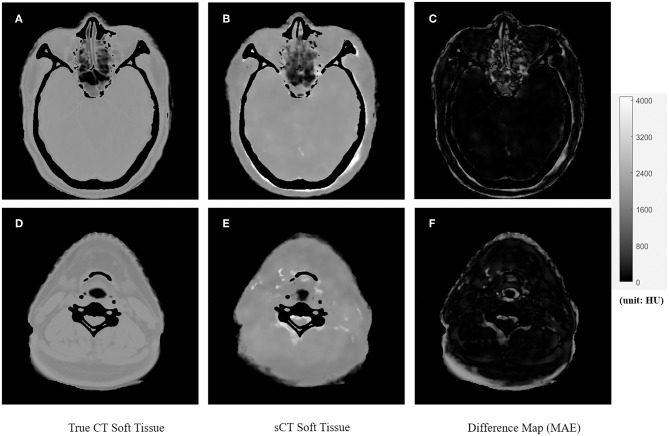
The comparison of true CT and synthetic CT for soft tissues. The first column is true CT images. The second column is the synthetic CT images. The third column is the difference maps. **(A–C)** Showed soft tissue in the head and nasal region. **(D–F)** Showed soft tissues in the neck region. Gray bar indicated the mapping from CT number to gray scale in the difference maps.

Figures 4, 5 showed the axial view for bone and soft tissues, respectively. The bone structures in synthetic CTs was well-reconstructed by our model, such as the nasal bone (Figure 4E) and bone marrow (Figures 4B,E). The soft tissues in synthetic CTs had the similar intensity as the real ones (Figures 5B,E). However, the interface between bone and soft tissues had higher deviation, and the delicate structures in nasal cavity were blurred in the synthetic CTs (Figure 5B). The majority soft tissue and bone region was reconstructed accurately except the interface between soft tissue and bone and some delicate structures in nasal cavity, where the inaccuracy might be induced by imperfect deformable registration.

### Quantitative Analysis

The summary of HU difference between the true CT and synthetic CT images was listed in Table 1. Compared to true CT, MAE of sCT in the 10 tested patients was 97 ± 13 HU in soft tissue, 131 ± 24 HU in overall region, and 357 ± 44 HU in bone, respectively. ME was −48 ± 10 HU in soft tissue, −6 ± 13 HU in overall region, and 247 ± 44 HU in bone, respectively. As shown in Table 1, MAE and ME varied in different patients. The synthetic CTs of Patient #1 had the lowest deviation in overall body, bone, and soft tissues (overall body: MAE = 91; bone: MAE = 300; soft tissue: MAE = 75; unit: HU). The synthetic CTs of Patient #3 had the largest deviations in overall body, bone, and soft tissues (overall body: MAE = 170; bone: MAE = 430; soft tissue: MAE = 118; unit: HU).

**Table 1:** Summary of all 10 test patients.

	**MAE: soft tissue**	**MAE: bone**	**MAE: overall region**	**ME: soft tissue**	**ME: bone**	**ME: overall region**
Patient 1	75	300	91	−32	191	−15
Patient 2	98	323	126	−58	178	−26
Patient 3	118	430	170	−49	312	15
Patient 4	109	370	145	−55	279	−8
Patient 5	79	303	100	−34	227	−6
Patient 6	104	369	136	−39	258	−1
Patient 7	93	343	120	−43	242	−9
Patient 8	93	421	158	−61	293	12
Patient 9	105	363	137	−50	274	−5
Patient 10	95	352	128	−57	217	−20
Mean ± SD	97 ± 13	357 ± 44	131 ± 24	−48 ± 10	247 ± 44	−6 ± 13

We also calculated ME to evaluate the average errors of each patient. In most patients (patient #1, 2, 4, 5, 6, 7, 9, 10), the synthetic CTs overestimated the CT number in the overall body region. Only in 2 patients (patient #3, 8), the CT number in synthetic CTs were underestimated, especially in the bone region. We noted that the CT number of bones in synthetic CTs was underestimated, while CT number of the soft tissues was overestimated using our U-net algorithm.

The GPU-based U-net model was trained with 23 patients' datasets using 20 h. The average time for each test patient was only 7 s. The total time of converting T2-weighted MRI to sCT for all 10 test patients using our deep learning algorithm was <1 min.

## Discussion

We have developed a feasible deep learning algorithm for converting MRI to HU maps to facilitate the MR-only treatment planning in the future. Based on the performance metrics such as MAE and ME, our soft tissue and overall region had acceptable HU differences. However, the bone region had larger errors due to less pixels of bone area compared to those of soft tissue and hence much less samples for training. In addition, bone regions have a large range of HU values, typically from several hundreds to several thousand HU numbers, which makes the training more difficult than the narrower range of HU numbers in soft tissue. One way to improve the results in the bone region is to separately train soft tissue and bone (30); another approach is to acquire ultrashort TE (UTE) MRI sequence to obtain better labeling of the bone region in MR images (31).

As mentioned in the previous review articles by Edmund et al. (15), there is no obvious favorable method among different types of MRI contrast(s) in the generation of synthetic CT to increase the accuracy. The reason we use the 2D images of T2-weighted MRI to generate synthetic CT images is simply due to its popularity in the existing radiotherapy workflow for target delineation. In our study, it took 20 h to train the U-net model with 23 patients' MRI and CT datasets. The average time for each test patient was only 7 s. The total time of converting T2-weighted MRI to sCT for all 10 test patients using our deep learning algorithm was <1 min, which has great clinical potential for online MRI conversion in the future.

It has been noticed by Edmund et al. (15) that the current performance metrics such as MAE and Dice do not reflect the corresponding dosimetric and geometrical agreement between the true CT and synthetic CT. Therefore, more unambiguous metrics should be developed, where the results should not depend on the selected CT number threshold (for example, our study used HU = 300 as the threshold for bone and soft tissue). Another concern of the synthetic CT methods is about the clinical implementation to the existing RT workflow. For the brain, it has been shown that a bulk density assignment may be sufficient for RT treatment planning (32). However, the head and neck region is more challenging in planning with many close-orientated organs at risk (OAR). Therefore, we may need more accurate HU maps in the conversion using the pixel-based deep learning method. We noticed there were underestimations in bones and overestimations in soft tissues. The use of L2 distance (mean squared error) as the loss function could cause the image blurring, which tended to predict an average CT value of both bone and soft tissues. The low prediction accuracy in the interface could be due to the errors of image registration and suboptimal prediction model. To encourage less blurring and improve the prediction accuracy, the L1 distance and a more complicated neural network with more fitting parameters could be introduced.

We have noticed several limitations in this study. First, the co-registration of MRI and CT-sim images may introduce systematic errors. It has been reported that MRI-CT co-registration may introduce geometrical uncertainties of ~2 mm for the brain and neck region (13) and of 2–3 mm for prostate and gynecological patients (14). Although our MRI and CT were acquired within the same week and similar scan position, the T2-weighted MRI was acquired in the department of diagnostic radiology without head and neck masks and without the flat couch top, the patients' chin position of CT-sim was still slightly different from that of MRI. Therefore, the rigid and deformable registration using the open source software could introduce geometrical errors, which makes the U-net downstream more difficult to accurately map HU values pixel-by-pixel. Furthermore, MRI has more geometrical distortion inherently compared to CT due to its gradient non-linearity and magnetic field inhomogeneity (33). In addition, patients inside the MRI bore can introduce geometrical distortion from susceptibility effect and chemical shift, which is difficult to correct. The traditional way of applying MRI to radiation treatment planning (RTP) is to acquire diagnostic MRI and then to conduct deformable image registration of MRI to the planning CT. The patient position of diagnostic MRI scans may be different from that of CT-sim or treatment position, which can introduce systematic errors (3, 34). Therefore, in order to minimize error and increase accuracy of deep learning-based MRI conversion to CT, we should use the MRI simulation with exactly the same immobilization devices as the CT simulation, which will be possible in 6 months when we have an in-department new MRI simulator.

The second limitation lies in the U-net deep learning algorithm. Deep learning algorithms are widely available, such as deep convolutional network (what we used), recurrent neural network (RNN), deep residual network (DRN), generative adversarial network (GAN), long/short term memory (LSTM). However, they may be susceptible to overfitting, difficult to interpret, or issues of accuracy. It has been reported that the deep CNN method competed favorably compared to the atlas-based method in the MRI conversion process (29). Here we used U-net CNN in the synthetic CT generation from T2-weighted MRI. However, U-net only interprets the non-linear mapping between MR and CT images through the training process. GAN, for example, has great potential to develop a better understanding of the non-linear relationship by generating images and improving the output through the discriminative algorithm (35). In the future, the structure of deep learning networks can be optimized to enhance accuracy and reduce the non-linear mapping error in the MRI conversion of CT numbers.

The third limitation is the sample size. It has been observed in our study that increasing the sample size can significantly improve the image quality and accuracy of the synthetic CT. For example, we started with 13 patients as the training set and later increased the sample size to 23 patients in the training set. The MAE of HU difference map has been decreased significantly. Also, we didn't use image augmentation to increase data sets in our study, which may help to improve the accuracy of MRI conversion to synthetic CT.

**Figure 6:**
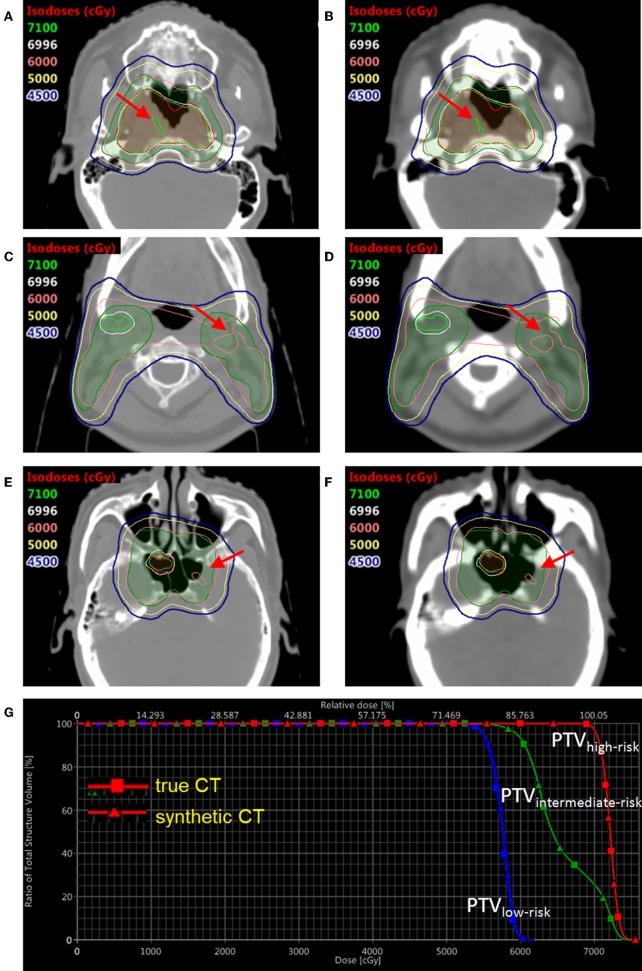
The dose distribution and DVH comparison between true CT and synthetic CT in an example NPC patient. Three slices of true CT are shown on the left **(A,C,E)**; Three corresponding slices of synthetic CT are shown on the right **(B,D,F)**. The DVH comparison based on true CT and synthetic CT is shown in **(G)**. The translucent red region is high-risk PTV with prescription dose of 69.96 Gy; the translucent green region is intermediate-risk PTV with prescription dose of 60 Gy; the translucent blue region (not shown in axial views here) is low-risk PTV with prescription dose of 54.4 Gy. DVH with squares and triangles is based on true and synthetic CT, respectively.

In order to compare the dose distribution using true vs. synthetic CT, one patient example was selected with the tumor in the sinus area and nearby fine bone structure (Figure 6). The mean HU value difference between true CT and sCT in the bone region was 191. The mean difference between true CT and sCT in the soft tissue region was 32. The treatment plan using the true CT was constructed with two full RapidArc in the Eclipse TPS v13.5 (Varian Medical Systems) and clinically approved by radiation oncologists. The dose distribution was subsequently recalculated based on sCT in the same treatment planning system. The three PTV regions, which were high-risk, intermediate-risk, and low-risk PTVs, as shown in DVH and isodose lines in Figure 6, had almost no difference between true and synthetic CT. For instance, the difference of D98% between the high-risk, intermediate-risk, and low-risk PTVs using true CT and sCT was <1%.

In summary, a promising method of synthetic CT generated from MRI has been proposed. Our pixel-based U-net deep learning algorithm of converting T2-weighted 2D MRI to HU mapping shows clinical potential of feasibility and simplicity with acceptable accuracy in soft tissue and overall region in the nasopharyngeal cancer site, which can be improved in the future by increasing the sample size of training data, acquiring same setup position of CT-sim vs. MRI-sim, and applying advanced neural networks such as GAN for better non-linear mapping.

## Data Availability Statement

All datasets generated for this study are included in the article/supplementary material.

## Ethics Statement

The studies involving human participants were reviewed and approved by IRB, CAMS Shenzhen Cancer Hospital. Written informed consent for participation was not required for this study in accordance with the national legislation and the institutional requirements.

## Author Contributions

YW conceived the idea, collected data, and wrote the manuscript. CL developed the U-net coding. XZ debugged the program and analyzed results. WD checked results and revised the manuscript.

### Conflict of Interest

The authors declare that the research was conducted in the absence of any commercial or financial relationships that could be construed as a potential conflict of interest.
